# Biotechnological Routes for Microplastic Mitigation: Current Challenges and Future Opportunities in the Enzymatic Degradation of Synthetic Textile Waste

**DOI:** 10.3390/polym18121419

**Published:** 2026-06-06

**Authors:** Aqsa Majeed, Diana Cayuela, Gabriela Mijas, Mauro Comes Franchini, Marta Riba-Moliner

**Affiliations:** 1 Department of Industrial Chemistry “Toso Montanari”, University of Bologna, Via P. Gobetti 85, 40129 Bologna, Italy; aqsa.majeed2@unibo.it (A.M.); mauro.comesfranchini@unibo.it (M.C.F.); 2 Terrassa Institute of Textile Research and Industrial Cooperation (INTEXTER), Universitat Politècnica de Catalunya BarcelonaTech (UPC), Campus Terrassa, Edif. TR7, C. Colom 15, 08222 Terrassa, Spain; diana.cayuela@upc.edu (D.C.); gabriela.mijas@upc.edu (G.M.)

**Keywords:** enzymatic recycling, synthetic textile fibre, biodegradation mechanisms, microplastic pollution, circular economy

## Abstract

The exponential growth of the global textile industry, largely driven by the demand for synthetic polymers such as poly(ethylene terephthalate) (PET), polyamides, and polyurethanes, has led to severe environmental consequences, notably the accumulation of persistent microplastics and solid waste. While conventional mechanical and chemical recycling methods are widely employed, they are often hindered by harsh processing conditions and the deterioration of material properties. Consequently, there is a critical need for sustainable end-of-life management strategies. This review provides a comprehensive analysis of the biodegradability of synthetic textile fibres, with a primary focus on emerging biotechnological and enzymatic recycling approaches. It systematically examines the intrinsic polymer characteristics that govern biodegradation—including molecular orientation, crystallinity, functional groups, and fibre chemistry—as well as extrinsic factors such as textile finishings, yarn twist, polymer blends, and chemical additives. Furthermore, the current landscape of microbial and enzymatic degradation routes is critically assessed, highlighting the specific mechanisms of biocatalysts (e.g., lipases, cutinases, PETase, and MHETase) in depolymerising complex synthetic matrices into recoverable monomers. Finally, this review identifies the existing literature gap between bulk plastic and textile-specific biodegradation, discussing future perspectives. By bridging polymer science and textile engineering, this work underscores the potential of enzymatic recycling to close the loop in synthetic fibre production and advance the transition toward a circular economy.

## 1. Introduction

For ages, the textile industry has been an integral part of human activity. Textile fabric production has experienced substantial growth over the past decades. Global output rose from 23.94 million metric tonnes in 1975 to 105.6 million metric tonnes by 2018—representing nearly a fourfold increase over a 40-year period [1,2]. This upward trend is projected to continue, with estimates suggesting an increase from approximately 116 million tonnes in 2022 to 147 million tonnes by 2030 [3].

Nowadays, this growing demand in the textile sector is largely met by synthetic fibres, which account for up to 64% of global textile fibre production. The remaining 36% consists of natural fibres, including cotton (24%), cellulosic fibres (6%), wool (1%), and other natural sources (4%) [4]. This distribution reflects current consumption patterns, where synthetic fibres—particularly polyester—dominate due to their cost-effectiveness, versatility, and performance characteristics [5].

However, the rising demand and consumption of synthetic textile products have intensified the environmental impacts associated with the textile industry, which is recognised as the third most polluted industry in the world, contributing to approximately 10% of the global carbon emissions [6]. Numerous studies have highlighted the environmental hazards posed by this sector, including contaminated wastewater, the release of toxic chemicals, and the emission of pollutants throughout the lifecycle of textile products that are generally incinerated and end up in landfills [7]. Unlike the natural homologues, which are derived from renewable and bio-based sources, synthetic fibres are typically non-biodegradable and primarily produced from petroleum-based sources and contribute substantially to environmental pollution, particularly through the release of persistent microplastics.

Microplastic pollution has become one of the most critical environmental concerns of the 21st century, with the textile industry identified as a major contributor. According to the European Environment Agency (EEA), it is estimated that between 200,000 and 500,000 tonnes of microplastics are released into the oceans each year from synthetic textiles alone, primarily during domestic laundering [8]. These microfibres are not only shed during washing but also throughout the entire life cycle of garments, including production, wear, and disposal.

The term microplastic refers to plastic particles smaller than 5 mm in their largest dimension, regardless of their shape (e.g., fragments, fibres, or spheres). These are generally classified into two categories: primary microplastics, which are intentionally manufactured at microscopic size (e.g., microbeads in cosmetics), and secondary microplastics, which result from the fragmentation of larger plastic items or the mechanical abrasion of synthetic textiles. In the latter case, the particles are often referred to as microfibres or microfibrils, although the terms are frequently used interchangeably in the literature [8]. According to the criteria established by the European Chemicals Agency (ECHA) [9] and adopted by the Spanish Ministry for the Ecological Transition and the Demographic Challenge (MITECO) [10], synthetic textile fibres released into the environment are classified under the broader category of synthetic polymer microparticles (microplastics). Specifically, the regulatory framework defines these entities as solid, non-biodegradable polymer particles with a fibre-like morphology, characterised by a length of ≤15 mm and a length-to-diameter ratio greater than 3. Consequently, in a regulatory and environmental context, the term ‘microfibre’ refers to a specific physical form of a microplastic pollutant rather than a distinct chemical category.

Recent studies estimate that textile-derived microplastics account for up to 35% of all primary microplastics in the marine environment, surpassing previously dominant sources such as personal care products or tyre wear. This shift is largely attributed to the increased global production of synthetic fibres, the proliferation of fast fashion, and the shortened lifespan of garments. While regulatory actions have significantly reduced the use of microbeads in cosmetics, textile microplastics remain largely unregulated, posing ongoing risks to aquatic ecosystems, soil quality, and potentially human health [8,11].

In parallel with the growing concern over microplastic emissions, attention has also turned to the end-of-life management of synthetic textiles. In response to the increasing production of synthetic fibres and the substantial volume of textile waste that is either incinerated or landfilled, various textile recycling methodologies have been developed. Despite considerable efforts, however, recycling remains a major challenge. Among the available strategies, chemical and mechanical recycling are the most widely employed [12,13].

Chemical recycling encompasses a range of processes—including pyrolysis, enzymatic hydrolysis, hydrothermal degradation, ammonolysis, gasification, and glycolysis—that contribute to the sustainable management of textile waste [14]. This approach has gained prominence in recent years, particularly for the treatment of synthetic fabrics, as it enables the depolymerisation of polymers into monomers, oligomers, or even raw gaseous or liquid forms. In some cases, re-polymerisation can be carried out to regenerate new polymers from these subunits, offering a potential circular solution for synthetic textile waste [15].

**Figure 1:**
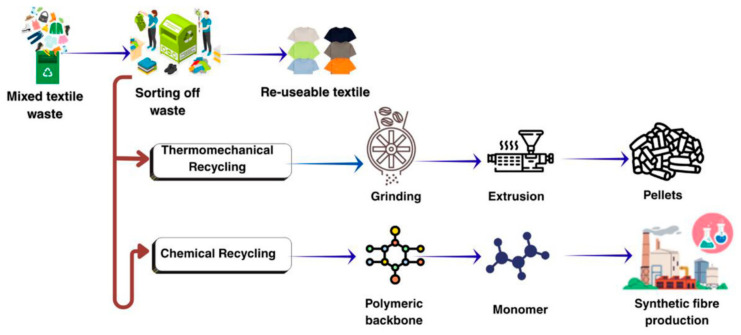
Schematic overview of textile waste recycling pathways: mixed textile waste is sorted into reusable materials or processed via thermomechanical recycling (grinding, extrusion, pellet formation) or chemical recycling (depolymerisation to polymer backbone and monomers) for synthetic fibre production.

On the other hand, mechanical recycling has been used since the early stages of the textile industry and is considered to be the most cost-effective and simplest recycling method [2,15]. In its strict sense, mechanical recycling involves the physical processing of textile waste, such as cutting, shredding, and fibre opening, to recover fibres that can be re-spun into yarns and used to produce new textiles. However, for thermoplastic synthetic fibres such as polyester, recycling typically involves thermomechanical processes, including melting and extrusion, rather than purely mechanical fibre recovery as shown in Figure 1 [16]. Both chemical and thermomechanical recycling methods present notable limitations: chemical recycling often requires harsh reaction conditions that hinder its large-scale application, while mechanical recycling tends to produce lower-quality fabrics with reduced mechanical performance [16,17]. In light of these limitations, there is a growing global shift toward more sustainable biotechnological approaches for the degradation and recyclability of textile materials. In biochemical recycling, enzymes and microorganisms are employed to break down polymers into their monomeric units. This process is considered environmentally friendly and, unlike chemical recycling, does not require aggressive chemical conditions. Enzymes act as biological catalysts, accelerating reaction rates and enhancing the efficiency of textile waste degradation [14].

Within the category of organic man-made fibres, a distinction is typically drawn between cellulosic and synthetic fibres. The enzymatic degradation of cellulosic materials has been extensively studied and is well understood—particularly due to the action of cellulases—synthetic fibres present a far more complex challenge. Building upon the growing environmental and technological concerns surrounding synthetic textile waste and microplastic pollution, this review aims to explore innovative strategies for textile recycling, with a particular focus on biological approaches. It examines the environmental challenges posed by synthetic textile waste, particularly the release of microplastics, and evaluates current recycling strategies. While thermomechanical and chemical recycling methods are widely known and successfully implemented in the industry, they present limitations in terms of economical investment, material quality and environmental impact. In response, there is increasing attention on biotechnological approaches that use enzymes and microorganisms to degrade synthetic polymers under mild conditions. This work provides a comparative analysis of these technologies, highlighting their feasibility, advantages, and limitations, and offers recommendations for future sustainable textile recycling solutions.

## 2. General Biodegradability Insights and Considerations

Synthetic fibres are man-made fibres produced through the polymerisation of monomers, typically derived from petrochemical sources. These polymers—such as polyesters, polyamides, polyacrylonitriles, and polyurethanes—are synthesised via step-growth or chain-growth polymerisation, resulting in macromolecules with high molecular weights and specific functional groups that determine their physicochemical behaviour. The chemical backbone of these polymers often includes aromatic rings, ester, amide, or nitrile groups, which contribute to their thermal stability, hydrophobicity, and resistance to microbial attack [18,19]. These parameters are critical to study and analyse in the development of processes aimed at enhancing the biodegradability of such materials.

This section examines the influence of specific structural and morphological parameters on the biodegradability of synthetic fibres. Factors such as fibre chemistry, molecular orientation and crystallinity, presence of finishings and other additives, blended fibres and influence of the fabric structure are considered, as they play a critical role in determining the accessibility of polymer chains to microbial and enzymatic degradation. The analysis focuses on synthetic fibres, whose biodegradation is commonly limited by durability- and performance-oriented design features.

### 2.1. Fibre Chemistry

The biodegradability of synthetic textile fibres is fundamentally governed by their chemical composition, particularly the nature of the functional groups present in the polymer backbone. These chemical groups determine the polymer’s susceptibility to hydrolysis, enzymatic cleavage, and microbial assimilation, which are essential steps in the biodegradation process.

**Figure 2:**
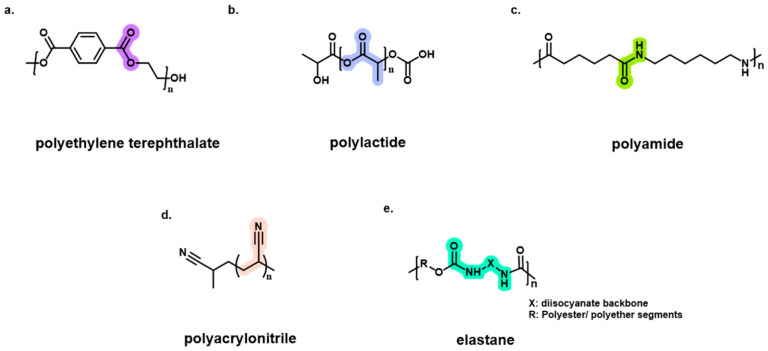
Representative polymeric backbone structures and functional groups (highlighted in colour) present (**a**) polyethylene terephthalate (PET), (**b**) polylactide (PLA), (**c**) polyamide (PA), (**d**) polyacrylonitrile (PAN), and (**e**) elastane (EL) fibres.

Fibres composed of aromatic polyesters, such as poly(ethylene terephthalate) (PET) (Figure 2a), contain ester linkages that are theoretically hydrolysable. However, the presence of aromatic rings adjacent to these ester groups increases rigidity and hydrophobicity, significantly reducing the accessibility of water and biological agents. This structural configuration contributes to PET’s high resistance to degradation under ambient conditions [20].

In contrast, aliphatic polyesters like polylactide (PLA) (Figure 2b) feature linear ester bonds without aromatic hindrance, making them more prone to hydrolysis. PLA is considered one of the most biodegradable synthetic fibres, especially under industrial composting conditions, due to its simpler and more accessible chemical structure [21].

Another example is polyamides, such as polyamide 6 (PA 6) and polyamide 6.6 (PA 6.6) (Figure 2c), that contain amide groups (-CONH-), which are also hydrolysable. However, the strong intermolecular hydrogen bonding between amide groups leads to high melting temperatures (215 °C and 265 °C for PA 6 and PA 6,6, respectively), higher per cent crystallinity and glass transition temperature (Tg), which limits water diffusion and slows down degradation rates. The biodegradability of polyamides is therefore lower than that of aliphatic polyesters, despite the presence of hydrolysable bonds [19].

On the other hand, polyacrylonitrile (PAN) (Figure 2d)-based fibres commonly found applications in acrylic textiles because of the presence of polar nitrile groups (-C≡N). The carbon–nitrogen triple bond possesses high bond dissociation energy and significant chemical stability, making it difficult for water molecules or hydroxyl ions to attack and cleave the polymer structure under normal environmental conditions. As the PAN polymeric backbone has adjacent nitrile groups, strong dipole–dipole interactions between these nitrile groups promote tight chain packing and high crystallinity, which reduces free volume, limits water diffusion, and restricts enzyme accessibility within the polymer matrix. All these factors contribute to making PAN one of the least biodegradable synthetic fibres [22,23].

Additionally, elastane (EL) (Figure 2e) fibres contain a mix of urethane and ether linkages, with biodegradability depending on the ratio of soft segments typically formed by polyether and polyester chains and hard segments formed by diisocyanate and chain extenders containing urethane linkages. This segmented structure gives the fibre selective mechanical strength, making the soft segments more accessible to degradation due to the presence of ester bonds, while the hard segments contribute to environmental persistence [24,25].

Thus, the type of chemical group, its position within the polymer chain, and the overall molecular architecture—including crystallinity, polarity, and hydrophobicity—play a decisive role in determining the biodegradability of synthetic fibres.

### 2.2. Molecular Orientation and Crystallinity

**Figure 3:**
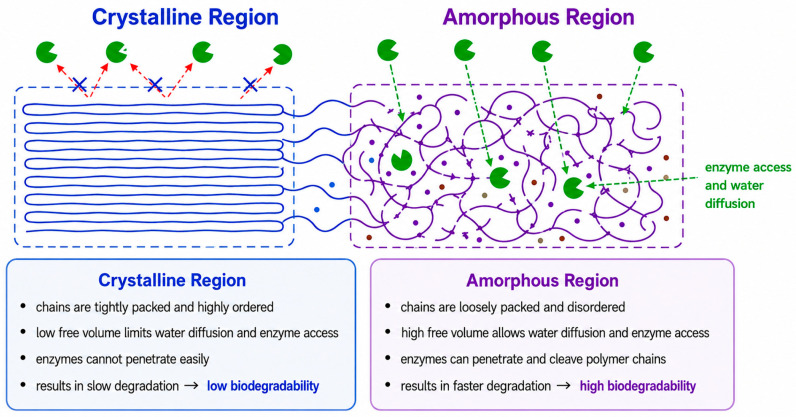
Schematic illustration of enzymatic degradation in amorphous and crystalline regions of polymer structures and their influence on biodegradability. Image generatively co-created with OpenAI, on 26 May 2026, from the prompt “make an illustration of woven, knitted and non-woven fabrics” [32].

To be considered as a fibre, a polymer must exhibit specific structural and mechanical characteristics. The defining feature of a textile fibre is its high aspect ratio-length significantly greater than diameter. Additionally, the polymer chains within the fibre must be oriented along the fibre axis, a property achieved through spinning techniques such as melt spinning [26], dry spinning [27] or wet spinning [28]. This molecular orientation enhances tensile strength, flexibility, and dimensional stability, distinguishing fibres from bulk plastics that lack such alignment. The internal structure of fibres typically includes both crystalline and amorphous regions, which influence properties like dyeability, moisture regain, and elasticity [18,29]. Also, macromolecular orientation of the chains enhances tensile strength and elongation at break in the direction of the fibre, making them more resistant to mechanical stress compared to isotropic bulk plastics. This high degree of orientation also contributes to increased crystallinity, which reduces the accessibility of enzymes and moisture, thereby impeding biodegradation [30,31]; meanwhile, amorphous zones are more susceptible to microbial attack. The proportion and distribution of these regions (crystalline and amorphous) vary by polymer type and processing conditions. For instance, fibres with higher amorphous content may degrade faster under composting or enzymatic treatment, whereas highly crystalline fibres like PET resist breakdown even under optimised conditions [30]. Figure 3 illustrates the effect of molecular orientation and crystallinity on biodegradability of synthetic fibres.

From the point of view of industrial processing, the processing history of synthetic fibres, encompassing the specific spinning conditions and subsequent thermomechanical treatments, is intrinsically linked to their final microstructure morphology. During the initial fibre-forming process, parameters such as the extrusion method, take-up speed, and cooling or coagulation dynamics induce significant alterations in the macromolecular arrangement. Furthermore, post-spinning processes, particularly drawing and thermal annealing, provide the necessary mechanical stress and activation energy for the polymer chains to reorganise into highly ordered, thermodynamically stable states. Consequently, these operational parameters dictate the fundamental microstructure of the fibre, directly defining both the overall degree of crystallinity and the extent of molecular chain orientation along the fibre axis.

The microstructure subsequently serves as the primary governing factor for the material’s chemical resistance and enzymatic accessibility. Whether assessing a fibre’s environmental persistence or its viability for advanced biocatalytic recycling, enzymatic action fundamentally relies on the ability of external agents, such as water and large enzyme molecules, to diffuse into the polymer matrix. While the loosely packed, unaligned amorphous regions of the fibre offer a readily accessible substrate for enzymatic attack and hydrolytic cleavage, the densely packed crystalline domains and highly oriented chain structures act as formidable steric barriers. Because bulky biocatalysts cannot readily penetrate these highly ordered structures, degradation is initially and primarily confined to the amorphous phases, making the precise ratio and distribution of crystalline to amorphous material critical.

For instance, fibres with higher amorphous content may degrade faster under composting or enzymatic treatment, whereas highly crystalline fibres like PET resist breakdown even under optimised conditions [30]. The dense packing of polymer chains and the presence of hydrophobic functional groups (e.g., aromatic rings in PET, nitrile groups in acrylics) limit water penetration and enzymatic hydrolysis [19]. Thus, the resistance to hydrolysis and microbial degradation leads to long-term persistence in the environment, contributing to the accumulation of textile waste and microplastic pollution.

Ultimately, the complex interplay between these structural characteristics can directly dictate the overall biodegradation kinetics and end-of-life breakdown profile of the fibrous material. Synthetic fibres subjected to high draw ratios and extensive thermal treatments typically exhibit elevated crystallinity and pronounced chain alignment; this results in severely restricted enzymatic accessibility and significantly prolonged degradation rates. Conversely, processing profiles that suppress crystallisation or limit molecular alignment yield predominantly amorphous structures that are far more susceptible to enzymatic hydrolysis. Therefore, the deliberate modulation of the processing history not only engineers the thermomechanical properties of synthetic fibres for their intended application, but also definitively sets the boundary conditions for their eventual biodegradation kinetics. As a direct consequence of this thermomechanical processing, synthetic textile fibres typically exhibit markedly lower degradation efficiencies compared to bulk plastic substrates, a recalcitrance driven by their elevated crystallinity and chain orientation and further compounded by their complex structural architectures and the presence of chemical surface finishes. While conventional recycling methods—mechanical and chemical—have been applied to synthetic fibres, they often suffer from limitations, as mentioned before.

### 2.3. Presence of Finishing and Other Additives

Synthetic textile fibres are frequently treated with a wide range of chemical finishes to enhance their performance, durability, and functionality. These finishes include flame retardants, water repellents, antimicrobial agents, and UV protectants, among others. While these treatments are essential for meeting industrial and consumer demands—such as safety, hygiene, and comfort—they can significantly hinder the biodegradability of the fibres by altering their surface chemistry and creating barriers to microbial and enzymatic degradation [31].

For instance, phosphorus-based flame retardants and halogenated compounds are commonly applied to polyester and polyamide fabrics used in protective clothing and upholstery. These compounds increase the thermal stability of the fibres but also reduce their susceptibility to hydrolysis and microbial colonisation. Similarly, durable water repellents (DWRs)—often based on fluorinated chemistries such as PFAS—form hydrophobic layers that prevent moisture penetration, thereby limiting the access of microorganisms and enzymes to the polymer surface [33,34].

Antimicrobial finishes, including silver nanoparticles, triclosan, and quaternary ammonium compounds, are widely used in sportswear, medical textiles, and home furnishings. While effective in inhibiting bacterial growth during use, these agents can also suppress microbial activity during the end-of-life phase, reducing the potential for biodegradation. Moreover, many of these finishes are persistent in the environment, contributing to long-term pollution and complicating waste treatment processes [35].

Experimental studies have demonstrated that fabrics treated with crosslinking agents or multifunctional coatings exhibit significantly lower biodegradation rates compared to untreated fabrics. For example, cotton fabrics treated with wrinkle-resistant and flame-retardant finishes showed up to 60% less weight loss and reduced CO_2_ evolution during soil burial tests, indicating limited microbial activity [36].

On the other hand, synthetic polymers are commonly modified during extrusion with a variety of chemical additives to enhance performance, processability, and durability. These additives are typically incorporated into the polymer melt prior to fibre formation and can significantly influence the fibre’s mechanical, thermal, and environmental behaviour. Additives play a crucial role in defining the performance and environmental behaviour of synthetic textile fibres. Synthetic polymers are commonly modified during extrusion with a variety of chemical additives to enhance performance, processability, and durability. These additives are typically incorporated into the polymer melt prior to fibre formation and can significantly influence the fibre’s mechanical, thermal, and environmental behaviour. While most conventional additives are designed to enhance durability, stability, and functionality, they often have unintended consequences on biodegradability [37]. Table 1 explains different type of additives and their functional importance and use in the textile industry.

Plasticisers, commonly added to polymers like chlorofibre (CLF) and elastane in order to increase chain mobility and impart flexibility to the polymer matrix while also increasing hydrophobicity and environmental persistence [46]. Flame retardants and UV stabilisers, essential for protective and outdoor textiles, enhance chemical resistance and reduce the likelihood of polymer breakdown under environmental stressors [39,47]. Similarly, antioxidants and antimicrobial agents inhibit oxidative and biological degradation, respectively, by stabilising the polymer matrix and suppressing microbial colonisation [48].

The presence of pigments and colourants introduces variability. While some inorganic pigments are inert, others may interfere with microbial activity or leach toxic compounds [47]. Nucleating agents, used to increase crystallinity, further reduce biodegradability by limiting the amorphous regions where enzymatic hydrolysis typically initiates [45].

### 2.4. Length and Twist of Yarns

There is another consideration to take into account. A yarn made of synthetic fibre can be presented as a continuous filament or a bunch of staple fibres that need to be processed to obtain yarns. Staple fibres are short-length fibres produced by cutting continuous filaments, and the choice between continuous filament and staple fibre formats depends on the intended textile application and environmental considerations. Continuous filaments offer superior mechanical strength and reduced microfibre shedding. In contrast, staple fibres provide greater bulk and softness, but require twisting during yarn formation, which can reduce biodegradability by limiting enzymatic access to the polymer chains. To transform these discrete fibres into usable yarns, a spinning process is required. This process involves several stages: opening and cleaning (blow room), fibre alignment (carding), drafting (draw frame), optional combing, roving, and finally spinning, where a twist is inserted to bind the fibres into a cohesive yarn [49]. Twist is a key structural parameter in yarn formation, responsible for binding the fibres together and imparting strength, flexibility, and abrasion resistance. The degree of twist affects the mechanical properties of the yarn, including tensile strength, elongation at break, and stiffness. Moreover, the act of twisting induces morphological changes that directly influence the biodegradation of the polymeric substrate: (i) reduces surface accessibility, (ii) increases crystallinity and orientation, (iii) compacts the microstructure, and (iv) causes twisted yarns to fragment differently than untwisted filaments [19,50].

### 2.5. Blended Fibres

Synthetic textile fibres are frequently used in blended formats, combining two or more fibre types to achieve enhanced performance characteristics in the final fabric. These blends are engineered to optimise properties such as durability, elasticity, softness, and thermal regulation. For example, cotton-polyester blends merge the breathability and softness of the natural fibre with the durability and wrinkle resistance of the synthetic component [51,52]. Similarly, polyamide is often blended with elastane and polyester to impart elastic recovery and abrasion resistance, particularly in activewear and hosiery applications [53].

Other notable blends include wool-polyester combinations, which enhance warmth and durability [45], and viscose-polyester blends, which improve drape and moisture management [46]. Acrylic fibres, known for their thermal insulation, are frequently blended with natural fibres such as wool to retain softness and warmth while improving resistance to shrinkage and stretching [54].

From a biodegradability standpoint, these fibre blends pose significant challenges. The presence of synthetic components, particularly those with high crystallinity and hydrophobicity (e.g., PET, polyamide), inhibits microbial degradation, even when blended with biodegradable natural fibres. The heterogeneous structure of blended fabrics complicates enzymatic access and reduces the efficiency of biological degradation pathways. Moreover, the interfacial bonding and physical entanglement between fibre types can obstruct separation processes, which are essential for targeted recycling and biodegradation [53].

Consequently, the separation of blended synthetic fabrics remains one of the most critical obstacles in textile waste management. Efficient recycling typically requires mono-material input, as most chemical and enzymatic recycling technologies are polymer-specific. The presence of multiple fibre types in a single textile product reduces the purity of recovered materials, increases processing costs, and limits the scalability of recycling systems [20,22].

### 2.6. Influence of the Fabric Structure

**Figure 4:**
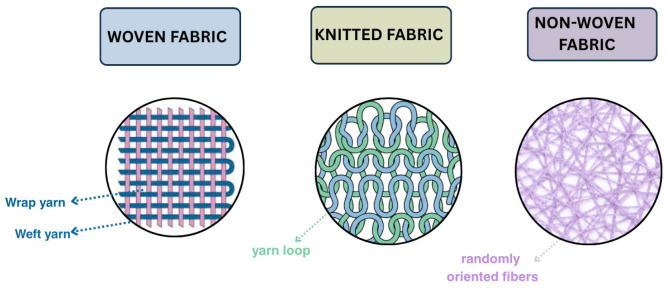
Schematic illustration of woven, knitted and non-woven fabric structures. From a general point of view, woven fabrics consist of two perpendicular sets of yarns (warp and weft) interlaced in an ordered pattern, resulting in a strong and dimensionally stable structure. Knitted fabrics are formed through interconnected loops of yarn, producing a flexible and stretchable structure with higher elasticity and porosity. In contrast, non-woven fabrics are composed of randomly or directionally arranged fibres bonded mechanically, chemically or thermally without traditional yarn interlacing, leading to a lightweight and less ordered structure.

Textile materials can be broadly classified into three main structural types: woven, knitted, and nonwoven fabrics (Figure 4), each characterised by distinct fibre arrangements and interlacing mechanisms. In addition, hybrid or composite structures combining these architectures—such as nonwoven–woven laminates or knitted–woven reinforcements—are increasingly used in technical and functional textiles. Within woven and knitted fabrics, the type of weave or knit structure (e.g., plain, twill, satin, rib, interlock) further modifies fabric compactness, pore size, and yarn exposure, all of which strongly influence biodegradation behaviour. Open and less compact structures, such as knitted jerseys or plain weaves with low thread density, promote greater water and oxygen diffusion and facilitate microbial colonisation. In contrast, compact ligaments such as twill or satin weaves, and tightly packed interlock knits, restrict fluid penetration and delay the onset of enzymatic or microbial attack. Some studies observed that loosely woven linen fabrics degraded faster than dense constructions when buried in soil, owing to higher surface area and moisture retention [55]. Similarly, a study on textile biodegradability highlighted that structural compactness and finishing treatments significantly affect environmental decay [43]. These findings emphasise that the biodegradation of polymeric textiles must be interpreted as a function of both molecular composition and fabric structure [45,56].

## 3. Current Scenario and Challenges

In the context of sustainable materials science, biodegradability refers to the capacity of a material to be broken down by biological agents—primarily microorganisms and enzymes—into simpler, non-toxic compounds such as water, carbon dioxide, and biomass [57].

**Figure 5:**
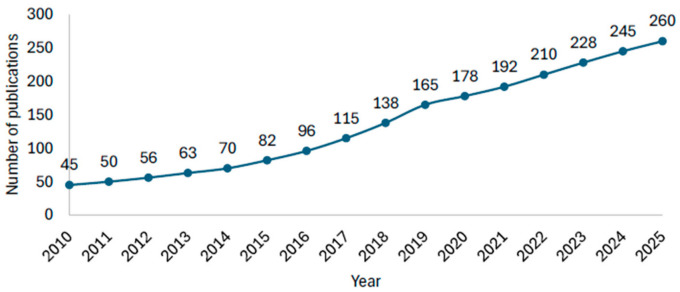
Evolution in scientific publications on plastic and textile fibre biodegradability (2010–2025) (extracted from Scopus and Web of Science data).

From 2010 to around 2015, the majority of scientific publications focused on plastic polymers, driven by global concerns over packaging waste and marine pollution. These studies explored microbial degradation, bioplastics, and enzymatic breakdown of conventional plastics such as polyethylene and polystyrene. In contrast, textile fibre biodegradability remained underrepresented until recent years. The complexity of textile structures, the use of blended materials, and the presence of functional finishes delayed systematic investigation. Evolution in scientific publications on plastic and textile fibre biodegradability (2010–2025) is shown in Figure 5. Nevertheless, since 2015, there has been a notable increase in textile-focused biodegradability research, reflecting growing awareness of microplastic release from laundering and the environmental impact of fast fashion. The convergence of publication trends in recent years suggests that textile biodegradability is becoming a priority, with research now addressing fibre morphology, additive effects and enzymatic recycling. This shift highlights the need for integrated approaches that consider both polymer chemistry and textile engineering in the development of sustainable materials (Figure 3). Recent publications such as those by Egan & Salmon (2021) [19] and Mehta (2023) [21] show a rapid increase in textile-focused biodegradability studies, indicating a shift in research priorities. This trend aligns with growing awareness of microplastic pollution from laundering and the environmental impact of fast fashion.

Biotechnological approaches offer a sustainable solution for recycling textile waste, particularly from synthetic fibres. In these systems, enzymes and microorganisms are employed as catalytic tools to accelerate polymer degradation and enhance fibre breakdown efficiency. The resulting monomers can be recovered and used as feedstocks for new polymer synthesis, thereby reducing the need for virgin resources. Ultimately, this biotechnological strategy contributes to closing the loop in synthetic fibre production and minimising waste generation [19].

Microbial biodegradation leverages the inherent capabilities of microorganisms such as bacteria and fungi to break down complex synthetic polymers into simpler compounds. Numerous microorganisms have been identified that produce enzymes capable of degrading synthetic fibres. In addition, researchers have investigated bioaugmentation and bio-stimulation techniques to enhance degradation efficiency [58,59]. Bioaugmentation involves introducing selected microbial strains into an environment, whereas bio-stimulation enhances the activity of native microbial communities by supplying nutrients or other growth-stimulating substances [60]. Recent advances in genetic engineering have enabled the modification of microorganisms to improve their capacity to degrade synthetic fibres. By incorporating specific genes or metabolic pathways, scientists can engineer microbial strains to more effectively break down complex polymer structures and convert them into valuable end products [19].

**Figure 6:**
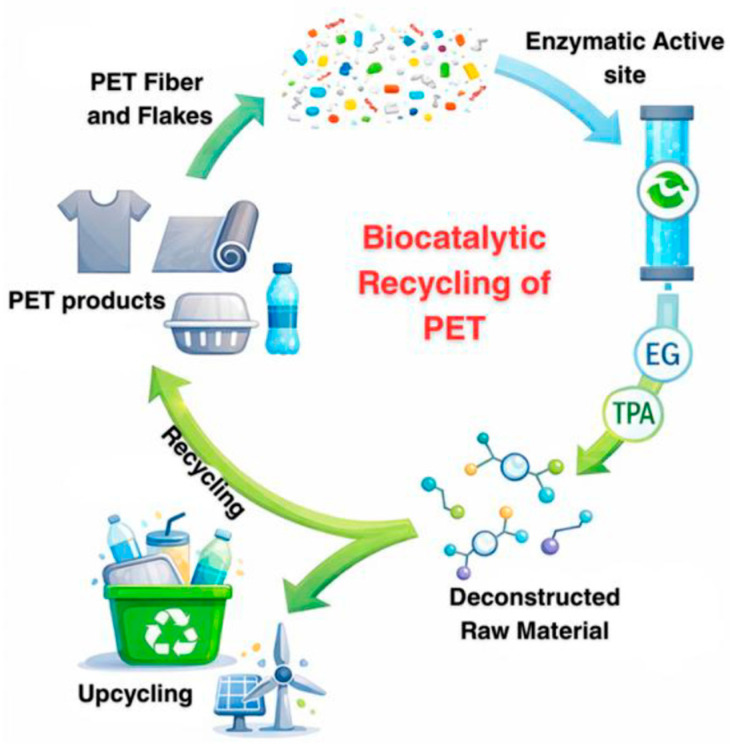
Schematic representation of the enzymatic recycling of poly(ethylene terephthalate) (PET), illustrating the conversion of PET waste (flakes and fibres) into monomers (terephthalic acid (TPA) and ethylene glycol (EG)) via an enzymatic platform, followed by recycling and upcycling into new products.

Enzymatic degradation of synthetic fibres relies on the targeted action of enzymes that cleave specific molecular bonds within polymer structures. These enzymes, also known as biocatalysts, are protein-based molecules produced by living organisms that accelerate chemical reactions. Enzymes such as proteases, lipases, and cellulases exhibit substrate specificity, enabling them to break peptide bonds (as found in polyamide, silk, and wool), ester bonds, and glycosidic bonds (as in cellulose), respectively. Through enzymatic hydrolysis, the fibre structure is broken down into shorter chains—oligomers, dimers, and eventually monomers—that can be utilised by microorganisms or serve as precursors for various biochemical processes. Beyond contributing to effective waste management, enzymatic degradation of synthetic fibres presents significant potential for advancing circular economy practices [53,58] as reported in Figure 6.

Hydrolytic degradation of the polymers is primarily mediated by the extracellular enzymes excreted by the microbial species. These microorganisms attach to the polymeric surface, initiating a heterogeneous enzymatic process. Enzymes typically interact with the substrate through a two-step mechanism. In the first step, enzymes are adsorbed onto the polymer surface, establishing close contact with the substrate. In the second stage, catalytic cleavage of covalent bonds occurs within the macromolecular backbone, leading to depolymerisation. This process may take place at the terminal ends (exo-attack) or within the internal segments of the polymer matrix (endo-attack) [53,61,62].

The degree of polymeric degradation is affected by the physical properties and chemical properties of the polymeric unit. The main factors that influence degradation are surface characteristics like area, hydrophilicity, and hydrophobicity, whereas enzymatic accessibility is controlled by first-order structural parameters like molecular weight and chemical structure. Degradation behaviour is also significantly influenced by higher-order properties such as modulus of elasticity, crystal structure, glass transition (Tg) and melting temperatures (Tm), and crystallinity [61,62]. Therefore, depending on the polymer structure and environmental conditions, degradation may proceed via two distinct mechanisms: surface erosion, where degradation is confined to the outer layers, and bulk degradation, where water permeates the interior, leading to uniform degradation throughout the matrix [63].

**Figure 7:**
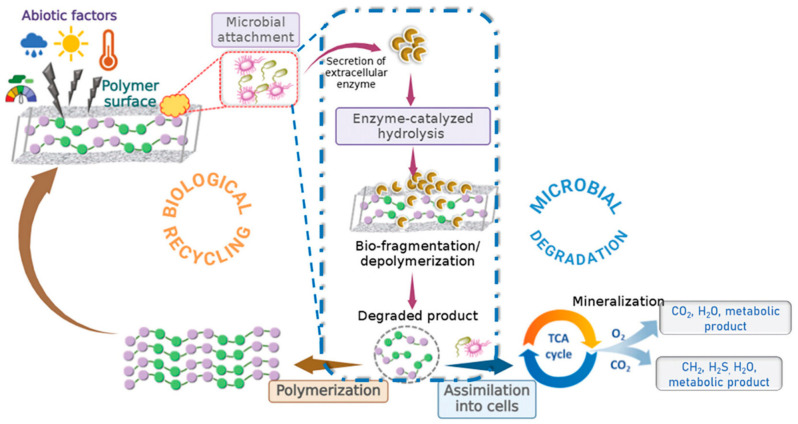
Mechanism of microbial degradation and biological recycling of enzymes (Reproduced from [53]).

The process of enzymatic hydrolysis usually occurs at the amorphous regions of the polymeric chain, which are more susceptible to enzymatic attack as compared to the highly ordered crystalline regions of the chain. The hydrolytic cleavage of polymer chains results in the release of low molecular weight fragments, such as oligomers and monomers, rendering enzymatic hydrolysis a spatially heterogeneous process. As degradation progresses, modifications in key physicochemical properties of the polymer—such as crystallinity, thermal stability, and polydispersity index—are often observed. These changes are closely associated with water diffusion into the polymer matrix, a process influenced by structural and morphological parameters including porosity, degree of crystallinity, surface roughness, hydrophobicity, and sample dimensions [63,64] (Figure 7).

### Biological Recycling

Although limited literature is available specifically for the recycling of textiles, enzymes are extensively studied and applied in the recycling of plastics, which often share the same backbone with synthetic fabrics. All the enzymes that are employed for this purpose predominantly belong to the hydrolases class of enzymes. Among them, cutinase, lipase and PETase are the most utilised, each demonstrating distinct structural characteristics and catalytic mechanisms. Table 2 shows the classification of enzyme families, their specific enzymes, and the synthetic polymers or textile fibres they degrade.

The enzymes, lipase and cutinase, belong to the esterases, which fall under the hydrolases class of enzymes [68]. Triacylglycerol acyl hydrolases, also known as lipases (EC 3.1.1.3), are common enzymes that are present in a variety of microbes, plants, insects, and animals. However, the main and most economical sources for industrial lipase synthesis are bacteria, yeasts, and fungi. Lipases, which belong to the hydrolase enzyme class, are essential for breaking ester bonds, especially those found in the carbon backbone of different polymers [67]. These enzymes transform triglycerides into glycerol and free fatty acids by catalysing their hydrolysis and synthesis [74]. Lipases stand out for their stability and catalytic efficiency in a wide variety of pH values and non-aqueous conditions without the need for cofactors. Depending on the substrate, they can be chemo-selective, regioselective, or stereoselective. Lipases are structurally characterised by a conserved catalytic triad consisting of a histidine, an acidic aspartate or glutamate residue, and a nucleophilic serine. Additionally, the catalytic serine is surrounded by a hydrophobic environment in its active site, creating an electrophilic area called the oxyanion hole. The presence of disulphide bridges also supports these enzymes’ catalytic activity and structural integrity [75].

Similarly, cutinases (EC 3.1.1.74) are serine esterases that belong to the α/β hydrolase superfamily and are primarily synthesised by plant pathogenic fungi, bacteria and plants. They are characterised by the presence of a conserved Ser-His-Asp catalytic triad and exhibit the properties that belong to both lipase and esterase enzymes, mainly hydrolysis, esterification, and transesterification reactions [66,76]. Cutinases are structurally different from lipases in that they do not have a hydrophobic “cap” over the active site, which exposes the catalytic serine residue to solvents and makes it easily accessible for substrate interaction [76]. Cutinases are particularly attractive for industrial applications due to their broad substrate specificity, capable of hydrolysing esters, polyesters, triacylglycerols, and waxes. This structural openness allows efficient catalysis of both low and high molecular weight esters, making cutinases highly versatile biocatalysts [77].

Within all types of cutinases, PETase and MHETase are the unique cutinase-like enzymes secreted by *Ideonella sakaiensis* 201-F6, a bacterium isolated from a plastic bottle recycling facility in Japan, which can utilise poly(ethylene terephthalate) (PET) as its sole carbon and energy source [78]. Structurally, PETase is distinguished by the presence of two disulfide bridges, unlike its other homologous enzymes that typically possess only one. This second sulphide bridge (DS2) connects the C-terminal loop to the final α-helix, enhancing its structural stability. Compared to previously identified PET-hydrolysing enzymes, PETase functions with a substantially greater catalytic efficiency under physiological circumstances (30 °C, pH 7.0), ranging from 5.5 to 120 times. MHETase then catalyses the hydrolysis of mono(2-hydroxyethyl) terephthalate (MHET) into bis(2-hydroxyethyl) terephthalate (BHET) and terephthalic acid (TPA). Despite sharing high sequence identity with other PET-hydrolysing cutinases, PETase’s superior performance suggests the presence of specialised structural elements that enhance substrate recognition and catalytic efficiency [65,79].

**Figure 8:**
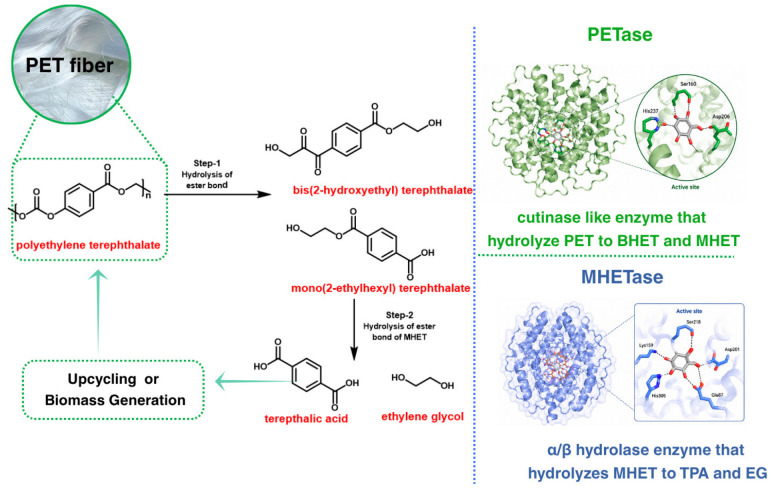
Schematic illustration of the biodegradation of polyethylene terephthalate (PET) fabric and the enzymes involved in its degradation pathway.

PETase and MHETase are not limited to plastic packaging but can be effectively applied to PET-based textile fibres, especially when combined with pretreatment methods that increase polymer accessibility. In this direction, a study conducted by Giraldo-Narcizo et al. demonstrated that ultraviolet ozone (UVO) pretreatment significantly enhances PETase-mediated degradation of cotton/PET blended textiles. They confirmed that alkali and UVO pretreatments improve PETase performance on commercial textile blends, with alkali treatment yielding the highest weight loss and UVO enhancing enzymatic TPA production. These results highlight the importance of pretreatment strategies to overcome the physical and chemical barriers posed by textile structures [80]. Figure 8 provides the schematic illustration of the process involved in the bio-degradation of PET.

Another study by Bhattacharya et al. focused on engineered PETase variants with improved thermal stability and catalytic activity. Although the primary substrate was amorphous PET, the findings suggest that these enhanced enzymes could be adapted for textile applications, especially where PET crystallinity is lower due to processing or wear [81].

Another fibre widespread is polyamide (PA 6 and PA 6.6), which presents significantly greater challenges than PET due to its intrinsic molecular structure and physical properties. Polyamides contain a high density of amide groups (-CONH-) that form strong intermolecular hydrogen bonds, resulting in highly crystalline regions. These ordered structures are thermodynamically stable and act as physical barriers, limiting enzyme accessibility. In contrast, PET has ester linkages with weaker hydrogen bonding and lower crystallinity, making it more susceptible to enzymatic hydrolysis. Amide bonds are chemically more stable than ester bonds due to resonance effects and partial double-bond character in the C–N linkage. This makes their enzymatic cleavage energetically less favourable. While enzymes like NylC hydrolase have been developed to target these bonds, their efficiency remains lower than that of PET-degrading enzymes such as PETase and MHETase. Nonetheless, Kato et al. [82] developed an enzymatic method to hydrolyse various aliphatic polyamides (including PA6 and PA6.6) using endo-type 6-aminohexanoate oligomer hydrolase (NylC, identified in Arthrobacter species). The enzyme was initially synthesised in an inactive form and underwent autocatalytic cleavage to become active. Structural analysis revealed a doughnut-shaped quaternary structure composed of αβ heterodimers, which was critical for substrate recognition and catalysis. Impressively, more than 80% of polymeric PA was converted into monomers under optimised conditions, demonstrating the enzyme’s potential for industrial-scale recycling of polyamide textiles.

On the other hand, Rietzler et al. [83] integrated green pretreatment strategies that improved enzyme accessibility by involving the reversible complexation and decomplexation of polyamide fibres using a CaCl_2_/ethanol/water (CEW) solvent system, enabling the non-destructive recovery of polyamides from mixed textile waste. This selective separation preserves the integrity of the fibres and facilitates subsequent enzymatic hydrolysis, making it a valuable complement to enzymatic systems like NylC.

However, other fibres like polyurethanes (PU), including elastane (EL), are considered one of the most challenging fibres to degrade due to their diverse chemical structure, which includes urethane, ether, and ester linkages, and often crosslinked networks [84]. A comprehensive review by Loredo-Treviño et al. [85] identifies esterases and proteases as the main enzymes capable of degrading PU. These enzymes are generally unspecific, acting on ester or amide bonds within the polymer matrix. Fungal enzymes, particularly from filamentous fungi, have also shown activity, although most studies focus on bacterial enzymes. A study by Peng et al. [86] demonstrated that *Pseudomonas putida* can degrade polyester-based polyurethane dispersion (Impranil DLN TM), achieving 92% degradation in 4 days. The degradation was confirmed via FTIR spectroscopy, showing a reduction in ester groups and emergence of amide groups. The responsible enzymes were found in both the extracellular medium and cytosol, with esterase activity confirmed in the cell lysate. Osman et al. [87] showed fungal-mediated degradation with *Aspergillus* sp. strain S45, isolated from soil. This was able to degrade PU films, with degradation more pronounced in the amorphous segments.

In contrast, polylactide (PLA) is a biobased, biodegradable thermoplastic polyester derived from lactic acid, typically produced via fermentation of renewable resources like corn or sugarcane. It is widely used in packaging, biomedical devices, and increasingly in textiles due to its mechanical properties comparable to PET and PA. PLA can degrade under industrial composting conditions and meets several standardised criteria for biodegradation, making it a promising alternative for sustainable textile applications [19,88]. PLA is compostable under industrial conditions, but its degradation in natural environments (e.g., soil, marine) is slow and incomplete due to its semi-crystalline structure and hydrophobicity. Biodegradation is enhanced under high temperature and humidity, such as in industrial composting and can be degraded enzymatically by several classes of enzymes: proteases, lipases, cutinases and estearases. These enzymes hydrolyse the ester bonds in PLA, releasing lactic acid as the main degradation product [89]. Enzymes from actinomycetes (e.g., *Actinomadura keratinilytica*) have shown high efficiency in degrading PLA powder, with up to 82% conversion [90]. A study by Lee & Song investigated the enzymatic hydrolysis of PLA fibres using lipases from various microbial sources. Optimal conditions (e.g., 40–45 °C, pH 7.5–8.0) led to measurable changes in tensile strength, thermal properties, and surface morphology, confirming enzymatic action on PLA textile fibres [91].

Finally, the biodegradability conditions of the main synthetic fibres in relation to the specific enzymes utilised are detailed in Table 3.

## 4. Future Perspectives

Although numerous studies have demonstrated the enzymatic degradation of synthetic polymers in their plastic form—such as films, pellets, or coatings—only a limited number have focused specifically on textile fibres. In some cases, results obtained from plastic substrates appear promising and suggest potential applicability to fibrous materials, especially when the polymers are in amorphous or low-molecular-weight forms. However, in the textile domain, the literature on enzymatic degradation of synthetic fibres remains scarce. This gap highlights the need for further research to evaluate enzyme accessibility, substrate specificity, and process optimisation in the context of fibre morphology, fabric structure, and textile finishing treatments, which may significantly influence degradability. Therefore, while enzymatic recycling of synthetic textiles is conceptually feasible, it is still in its early stages and requires substantial development to reach practical implementation.

An emerging research direction in the field of synthetic fibre biodegradation is the incorporation of additives or co-monomers designed to facilitate subsequent enzymatic degradation. These additives may act by reducing polymer crystallinity, increasing hydrophilicity, or introducing labile bonds that are more susceptible to enzymatic hydrolysis. For example, the inclusion of ester-containing soft segments or hydrolysable linkers in polyamide or polyurethane formulations could enhance enzyme accessibility and activity. Additionally, pro-degradant additives—commonly used in oxo-degradable plastics—might be adapted to textile-grade polymers to initiate partial chain scission, thereby improving the efficiency of enzymatic attack. Although this strategy has been explored in the context of plastic packaging, its application to textile fibres remains largely unexplored and represents a promising avenue for future research aimed at enabling controlled biodegradation of synthetic textiles. In this direction, CiCLO^®^ is a textile additive that is incorporated into synthetic fibres during manufacturing. It creates biologically accessible sites within the polymer matrix, mimicking the behaviour of natural fibres like wool. These sites allow microorganisms to recognise and metabolise the synthetic material more easily, leading to biodegradation in environments where traditional synthetics would persist for decades. Unlike compostable bioplastics (e.g., PLA), CiCLO^®^-treated fibres are designed to degrade in anaerobic landfill or marine environments, not just industrial composting facilities. According to the review by Egan & Salmon [19], technologies like CiCLO^®^ represent a promising strategy for improving the biodegradability of synthetic fibres, although independent peer-reviewed studies on long-term environmental impact and degradation kinetics are still limited.

Another promising strategy to improve the enzymatic degradation of synthetic textile fibres is the use of pre-treatment methods that modify the polymer structure to make it more accessible to enzymes. Synthetic fibres such as PET, polyamide, and polyurethane often exhibit high crystallinity, strong hydrogen bonding, and hydrophobic surfaces, which limit enzyme penetration and activity. Pre-treatments can help overcome these barriers by reducing the crystallinity (e.g., via thermal or chemical treatment), increasing the surface area and porosity (e.g., through mechanical milling or plasma treatment), introducing functional groups that enhance hydrophilicity or serve as enzyme binding sites, and partially depolymerising the polymer chains, making them more susceptible to enzymatic hydrolysis. Also, pre-treatments could be integrated into finishing processes, such as enzymatic scouring, ozone treatment, or low-temperature plasma, which are already used to modify fibre surfaces. These could be adapted to enhance enzymatic accessibility for recycling purposes. Boondaeng et al. (2023) [107] investigated the enzymatic hydrolysis of cotton/PET textile blends using a reusable alkaline pre-treatment with 15% NaOH solution and temperature. They effectively disrupted the fibre matrix, enabling subsequent enzymatic hydrolysis with cellulase to reach a glucose yield of 89.7% after 96 h. On the other hand, Durur and Varan (2018) [108] applied glow discharge plasma to PET and PET/elastane fabrics. Although the study focused on hydrophobic coatings and surface energy, the plasma treatment was shown to alter the surface sufficiently to suggest potential for improved enzymatic hydrolysis.

Although pyrolysis is traditionally associated with high-temperature thermal decomposition, recent studies have investigated low-temperature pyrolysis (LTP) as a means to modify polymeric substrates and enhance their susceptibility to enzymatic hydrolysis. This approach aims to partially depolymerise or oxidise the polymer backbone, reduce crystallinity, and expose functional groups that facilitate enzymatic attack. For example, Taher et al. (2024) [109] studied the pyrolysis behaviour of PET plastic waste in the presence of activated montmorillonite (AMMT) as a catalyst. The treatment reduced the onset and maximum decomposition temperatures of PET and altered the distribution of evolved gases. Although the study focused on energy recovery, the reduction in carbonaceous residue and increased surface reactivity suggest potential for subsequent enzymatic treatment. However, while pyrolysis is well-established for energy recovery and monomer production, its role as a pre-treatment for enzymatic recycling of textile fibres is still in its infancy. The lack of studies directly combining LTP with enzymatic hydrolysis of PET or polyamide textiles suggests a valuable opportunity for future research. Optimising pyrolysis conditions to avoid excessive carbonisation while enhancing enzymatic accessibility could open new pathways for hybrid recycling strategies.

Thus, the durability and chemical resistance imparted by these finishes, while beneficial during the product’s lifecycle, pose substantial challenges for biological recycling and composting. As such, the development of eco-friendly and biodegradable finishing agents (such as bio-based repellents, non-metal antimicrobials, and formaldehyde-free crosslinkers) is gaining attention as a strategy to align textile performance with environmental sustainability.

## 5. Conclusions

The study of biodegradability is not only essential for evaluating the environmental footprint of textile waste, but also for informing the design of future fibres that are compatible with circular economy principles. As the textile industry faces increasing pressure to reduce its ecological impact, understanding the interplay between polymer chemistry, fibre morphology, and additive formulations becomes critical for enabling effective biological recycling strategies.

Enzymatic recycling has shown promising results in the degradation of synthetic polymers such as PET and polyamides, especially when combined with enzyme engineering, microbial consortia, and process optimisation. These advances have challenged the notion that such polymers are inherently non-biodegradable. However, textiles present unique challenges compared to packaging or bulk plastics. Their structural complexity, variability in fibre formats (e.g., filaments, staple fibres, nonwovens), and the presence of functional finishes, dyes, flame retardants, and other additives can significantly hinder enzymatic access and activity.

One of the most critical barriers to enzymatic recycling is the widespread use of fibre blends, particularly those combining natural and synthetic components. These blends are difficult to separate mechanically and often require different enzymatic pathways for degradation. The lack of standardised labelling and transparency regarding fibre composition and chemical treatments further complicates sorting and process design. Therefore, eco-design strategies that prioritise mono-material constructions, minimise additive complexity, and incorporate clear labelling systems are essential to facilitate future recycling efforts.

To overcome the biochemical limitations posed by textile substrates, researchers are exploring the use of enzyme cocktails—combinations of enzymes with complementary activities that can act synergistically on complex polymer matrices. Additionally, pre-treatment techniques such as mechanical disruption, thermal conditioning, or mild chemical hydrolysis may enhance substrate accessibility and improve enzymatic efficiency. The integration of enzymatic recycling with other technologies, such as solvent-based separation or selective depolymerisation, could offer hybrid solutions tailored to specific textile waste streams.

Despite the technical and logistical challenges, enzymatic recycling holds great promise as a low-energy, selective, and potentially scalable alternative to conventional methods. Its specificity allows for targeted depolymerisation without generating toxic by-products, and its adaptability opens the door to customised solutions for diverse textile materials. However, transitioning from laboratory-scale success to industrial implementation will require significant investment in biotechnological infrastructure, regulatory support, and cross-sector collaboration.

In conclusion, while enzymatic recycling of textiles is still in its infancy, the convergence of biotechnology, materials science, and sustainable design offers a hopeful trajectory. Continued research, pilot-scale demonstrations, and policy frameworks that incentivise recyclable fibre design and transparent labelling will be key to unlocking the full potential of this approach. With coordinated efforts across academia, industry, and government, enzymatic recycling could become a cornerstone of sustainable textile waste management—transforming a complex challenge into a viable opportunity for circular innovation.

## Figures and Tables

**Table 1:** Overview of common additives used in textile fibres, their functional roles, typical applications, and their impact on biodegradability, including the underlying mechanisms affecting microbial and enzymatic degradation.

Additive	Function/Use	Substrate Applied	Impact on Biodegradability ^1^	Mechanism of Impact	Reference
Plasticizers	Increase flexibility, reduce brittleness	CLF, EL, some acrylics	Negative	Increase hydrophobicity and persistence; may leach and resist microbial degradation	[38]
Flame retardants	Improve fire resistance	PET, PA, EL	Negative	Enhance chemical stability; reduce microbial and enzymatic accessibility	[39,40]
UV stabilisers	Prevent photodegradation	Outdoor PET, PA	Negative	Protect polymer chains from UV-induced breakdown, limiting degradation	[41]
Antioxidants	Prevents oxidative degradation during processing	All synthetic fibres	Negative	Stabilise polymer chains, reducing susceptibility to oxidative and enzymatic attack	[42,43]
Antimicrobial agents	Inhibit microbial growth	Sportswear, medical textiles	Negative	Suppress microbial colonisation, limiting biodegradation on initiation	[43]
Pigments/colorants	Provide colour and aesthetic properties	All fibres	Variable	Some are inert; others may interfere with microbial activity or leach toxic compounds	[44]
Nucleating agents	Promote crystallinity	PET, PLA	Negative	Increase crystalline regions, reducing enzymatic access	[45]

^1^ A negative or positive impact on biodegradability means that its presence in the fibre reduces/inhibits or enhances the ability of the polymer to break down, respectively.

**Table 2:** Overview of key enzyme families involved in polymer degradation, highlighting representative enzymes and their specificity toward different synthetic polymers and textile fibres.

Enzyme Family	Specific Enzyme	Target Polymer/Fibre ^1^	Reference
Hydrolase	PETase	PET	[65]
	Cutinase	PET, PLA, EL	[66]
	Lipase	EL, PLA	[67]
	Esterase	PLA, EL	[68]
Oxidoreductase	Laccase	PE, PP, PS	[69]
	Peroxidase		[70]
Other enzymes	Dehalogenase	CLF	[71]
	Carbonate hydrolase	PC	[72]
	Monooxygenase	Synthetic rubber	[73]

^1^ PET = poly(ethylene terephthalate), PLA = polylactide, EL = elastane, PE = poly(ethylene), PP = poly(propylene), PS = poly(styrene), CLF = chlorofibre, PC = poly(carbonate).

**Table 3:** Detailed summary of the biodegradability conditions of the main synthetic fibres according to the type of enzyme used.

Substrate	Enzyme	Temperature (°C)	Time	Results	Reference
PET	PET hydrolase LCC^ICCG^	72	23 h	31.2% degradation of the polymeric surface	[92]
	Lipase (*T. lanuginosus*)	37	120 h	~7-fold increase in hydrolysis products released (with Triton X-100) vs. no detergent; lipase shows increase in dyeability (surface polar groups) in presence of plasticizer DEPA	[93]
	Cutinase (*T. fusca*)	60	120 h	Increased hydrolysis rates in presence of plasticizer DEPA; improved dye ability/colour depth enhancement: ~300% for lipase, ~130% for cutinase in presence of plasticizer vs. without	[93]
	Cutinase (*F. solani*)	37	120 h	Increased hydrolysis rates in presence of plasticizer DEPA; improved dye ability/colour depth enhancement: ~300% for lipase, ~130% for cutinase in presence of plasticizer vs. without	[93]
	Cutinase (*H. insolens)*	55	24 h	30 ± 2% yield of terephthalic acid (TPA) from the PET component	[94]
		50	24 h	97% pure terephthalic acid (TPA)	[95]
	Cellulase	55	24 h	83 ± 4% yield of glucose from cotton component, without reducing the TPA yield	[94]
	Cutinase (*T. fusca*)	60	120 h	Released ~50× more degradation products from amorphous than from semicrystalline fibres.	[96]
	Lipase (*T. lanuginosus)*	37	120 h	Released about twice as much MHET and TA from amorphous fibres; small amounts of BHET detected only in amorphous samples	[96]
Acrylic	Cutinase (*F. solani*)	30	3 h	30% increase in the colour depth because of more functional group available	[97]
	Esterase (Texazym PES)	30	3 h	25% increase in the colour depth	[97]
	Nitrile hydratase	20–25	10 min	Conversion of nitrile groups into the corresponding amides	[98]
	Nitrilase	40	2 to 36 h	Conversion of nitrile groups into the corresponding carboxylic acids, release of ammonia and poly-acrylic acid	[99]
	Nitrile hydratase (*C. nitrilophilus*) and cutinase	30	3 h	Increased surface hydrophilicity, wettability and dye-ability because of release of functional groups on the surface	[100]
PA6.6	Esterase	Room temperature	1 h	Limited hydrolysis of the polyamide surface and release of adipic acid	[101]
	Protease	Room temperature	1 h	Limited hydrolysis of the polyamide surface and release of adipic acid	[101]
	Amidase	Room temperature	1 h	Limited hydrolysis of the polyamide surface and release of adipic acid	[101]
	Cutinase (*F. solani*)	37	4 h	Higher agitation enhanced enzymatic hydrolysis, resulting in increased amino acid release and improved dye uptake	[102]
	Protease (*Bacillus* sp.)	35	4 h	Fivefold increase in amino group release as compared to cutinase; dye uptake also enhanced	[102]
	Protease + Lipase	40	90 min	Increased release of amine groups and cleavage of peptide bonds	[103]
	Protease (*B.licheniformis*)	60	1 h	Increased hydrophilicity, smoother surface of the fabric	[104]
	Lipase	30	80 min	Enzymatic hydrolysis of PA chains and amide functional groups improved wettability and dyeability	[105]
	Nitrilases or nitrile hydratases/amidase (*M. lutues* BST20)	60	24 h	Hydrolysation of the nitrile groups into the corresponding acid, release of ammonia	[106]

## Data Availability

No new data were created or analysed in this study.
